# Endothelial Activin Receptor-Like Kinase 1 (ALK1) Regulates Myofibroblast Emergence and Peritubular Capillary Stability in the Early Stages of Kidney Fibrosis

**DOI:** 10.3389/fphar.2022.843732

**Published:** 2022-06-13

**Authors:** Carlos Martínez-Salgado, Fernando Sánchez-Juanes, Francisco J. López-Hernández, José M. Muñoz-Félix

**Affiliations:** ^1^ Department of Physiology and Pharmacology, Translational Research on Renal and Cardiovascular Diseases (TRECARD)-REDINREN (ISCIII), University of Salamanca, Salamanca, Spain; ^2^ Institute of Biomedical Research of Salamanca (IBSAL), Salamanca, Spain; ^3^ Department of Biochemistry and Molecular Biology, University of Salamanca, Salamanca, Spain

**Keywords:** ALK1, Angiogenesis, peritubular capillaries, myofibroblasts, fibrosis, chronic kidney disease

## Abstract

Renal tubulo-interstitial fibrosis is characterized by the excessive accumulation of extracellular matrix (ECM) in the tubular interstitium during chronic kidney disease. The main source of ECM proteins are emerging and proliferating myofibroblasts. The sources of myofibroblasts in the renal tubular interstitium have been studied during decades, in which the epithelial contribution of the myofibroblast population through the epithelial-to-mesenchymal (EMT) process was assumed to be the major mechanism. However, it is now accepted that the EMT contribution is very limited and other mechanisms such as the proliferation of local resident fibroblasts or the transdifferentiation of endothelial cells seem to be more relevant. Activin receptor-like kinase 1 (ALK1) is a type I receptor which belongs to the transforming growth factor beta (TGF-*β*) superfamily, with a key role in tissue fibrosis and production of ECM by myofibroblast. Predominantly expressed in endothelial cells, ALK1 also plays an important role in angiogenesis and vessel maturation, but the relation of these processes with kidney fibrosis is not fully understood. We show that after 3 days of unilateral ureteral obstruction (UUO), ALK1 heterozygous mice (*Alk1* ^*+/−*^) display lower levels of kidney fibrosis associated to a lower number of myofibroblasts. Moreover, *Alk1* ^*+/−*^ mice have a lower degree of vascular rarefaction, showing improved peritubular microvasculature after UUO. All these data suggest an important role of ALK1 in regulating vascular rarefaction and emergence of myofibroblasts.

## Introduction

Tissue fibrosis is a common process to several chronic diseases of the liver, lungs, and kidneys, characterized by loss of tissue parenchyma (hepatocytes, pneumocytes and tubular epithelial cells, respectively), abundance of myofibroblasts, increased secretion of extracellular matrix proteins (ECM) and capillary rarefaction (Zeisberg and Kalluri, 2013). Specifically, in chronic kidney disease (CKD), a progressive and irreversible loss of renal function and renal tissue integrity, is associated with tubulo-interstitial fibrosis resulting from excessive deposition of ECM proteins by myofibroblasts. Different sources of myofibroblasts contribute to renal fibrosis (Grande and López-Novoa, 2009; Munoz-Felix and Martinez-Salgado, 2021). During years, the epithelial-to-mesenchymal transition (EMT) was considered the main source (Sato et al., 2003; Zeisberg et al., 2003; Grande and López-Novoa, 2009; Grande et al., 2010) (of myofibroblasts). However, it was demonstrated that the epithelial contribution to myofibroblast abundance was around 5%, and other mechanisms such as the proliferation of local resident fibroblasts, the endothelial-to-mesenchymal transition (EndMT) and other mechanisms were involved (LeBleu et al., 2013; Grande et al., 2015).

Loss of the renal microcirculation due to blood vessel dropout is a major feature of chronic kidney disease (CKD) which also correlates with the progression of renal injury and tissue regeneration (Ishii et al., 2005). The loss of peritubular capillaries (PTC) also correlates with hypoxia and the development of fibrosis (Goligorsky, 2010; Gewin, 2019). Multiple mechanisms contribute to microvascular rarefaction such as “drive in reverse” or anti-angiogenic reprogramming due to the induction of anti-angiogenic programs promoted by angiostatin or endostatin (Goligorsky, 2010). In renal fibrosis, PTC undergo rarefaction after kidney injury (Kida et al., 2014). In an early phase, angiogenic factors are upregulated, endothelial cells proliferate and pericytes migrate away from the capillary area. Subsequently, a progression phase ensues with vascular regression, endothelial cell disfunction and apoptosis (Kida et al., 2014).

Activin receptor-like kinase 1 (ALK1) is a type I receptor from the TGF-*β*1 superfamily with a documented role in regulating ECM deposition and thus tissue fibrosis in the skin (Morris et al., 2011), liver (Breitkopf-Heinlein et al., 2017; Desroches-Castan et al., 2019a), heart (Morine et al., 2017a) and kidneys (Muñoz-Félix et al., 2014a). We previously showed that the increased renal fibrosis associated to ALK1 heterozygosity after 15 days of unilateral ureteral obstruction (UUO) was due to the promotion of ECM protein synthesis in myofibroblasts, the major source of fibrotic matrix (Muñoz-Félix et al., 2014a). ALK1 is also involved in the regulation of endothelial cell activation (Lamouille et al., 2002; Jonker, 2014), which impinges on vascular homeostatic processes. ALK1 seems to have a dual role in angiogenesis. While some studies show a pro-angiogenic role (Goumans et al., 2002; Lebrin et al., 2005), some others have demonstrated that ALK1 inhibits the activation phase of angiogenesis (Lamouille et al., 2002; Larrivée et al., 2012), especially when activated by its high affinity ligand bone morphogenetic protein 9 (BMP9), a quiescent factor promoting the normalization of the vasculature (David et al., 2007a; David et al., 2008; Ouarné et al., 2018; Viallard et al., 2020). Apart from our previous studies, it has been recently shown the protective role of ALK1 in diabetic nephropathy due to its effect in blood vessel integrity maintenance (Lora Gil et al., 2020; Gil et al., 2021). Yet, the contribution of alterations in vascular homeostasis to tissue fibrosis is not completely understood.

In this manuscript we aim to elucidate the role of ALK1 in renal vascular rarefaction and integrity in a fibrotic scenario produced by UUO.

## Materials and Methods

### Mice

We used ALK1 heterozygous mice to evaluate the role of ALK1 in the early changes of the ureteral obstruction. *Alk1* ^*+/−*^ mice were generated as previously described (Oh et al., 2000). Adult *Alk1* ^*+/−*^ mice were kept in the pathogen-free facilities for genetically modified mice of the Animal Experimentation Service, University of Salamanca. Genotype analysis was performed by PCR with DNA isolated from mouse tail biopsies and using the primers previously reported (Oh et al., 2000).

### *In vivo* Experimental Model of Tubulointerstitial Fibrosis

Unilateral ureteral obstruction (UUO) is an experimental model of renal injury, which causes tubular cell injury, inflammation and fibrosis. UUO has been used as a model for the events that take place during chronic kidney disease (Ucero et al., 2014).

UUO was performed during 3 days, as we aim to evaluate the early changes of this experimental approach. The unilateral ureteral obstruction (UUO) was performed as previously described (Rodríguez-Peña et al., 2002; Grande et al., 2009). In brief, 8 weeks old male mice were anesthetized with Isoflurane (Schering-Plough, Madrid, Spain). After laparatomy, we used non-reabsorbable 5-0 silk to ligate the left ureter. To generate sham operated mice (SO), we manipulated the left kidney ureter without ligation.

In this study, 5 mice were included in each experimental group. Animals were kept under controlled ambient conditions in a temperature controlled-room with a 12 h light/dark cycle, and were reared on standard chow (Panlab, Barcelona, Spain) and water *ad libitum*. In all procedures, mice were treated in accordance with the Recommendations of the Helsinki Declaration on the Advice on Care and Use of Animals referred to in: law 14\/2 007 (3 July) on Biomedical Research, Conseil de l´Europe (published in Official Daily N. L358/1-358/6, 18-12-1986), Government Spanish (Royal Decree 223/1 988, (14 March) and Order of 13-10-1989, and Official Bulletin of the State b. 256, pp. 31349-31362, 28-10-1990). The procedure was approved for the Bioethics committee of the University of Salamanca and Consejería de Agricultura y Pesca (Junta de Castilla y León).

### Renal Tissue Preparation

Obstructed (O) and contralateral kidneys (NO), as well as kidneys of sham operated mice (SO), were removed 3 days after surgery after perfusion with heparinized saline solution at 37°C in order to eliminate red blood cells from the tissue and to avoid endogenous peroxidase signals in immunohistochemistry procedures. Next, kidneys were halved longitudinally in order to use one half for protein extraction and analysis and the other half for stainings and immunohistochemistry. Renal samples for protein extraction were frozen in liquid nitrogen and stored at −80°C. Renal samples for histological studies were fixed for 24 h in formaldehyde, transferred to ethanol 70% and then embedded in paraffin.

### Western Blot

Western blot was performed for protein levels analysis in mouse kidney tissue. Tissue protein extracts were homogenized in lysis buffer (150 mmol/L NaCl, 1% Igepal CA-630, 10 mmol/L MgCl_2_, 1 mmol/L EDTA, 10% glycerol, 1 mmol/L Na_3_VO_4_, 25 mmol/L NaF, l mmol/L PMSF, 10 mg/ml aprotinin and 10 mg/ml leupeptin) containing 25 mmol/L HEPES, pH 7.5 and centrifuged at 14000 g during 20 min at 4°C. Supernatants were recovered and the protein concentration was quantified using Lowry Method (BioRad). Lysates (100 µg per lane) were loaded onto polyacrylamide gels and the proteins were transferred to PVDF membranes (Millipore, Billerica, MA, United States) by electroblotting. Next, membranes were blocked in bovine serum albumin (BSA) 3% and were incubated overnight at 4°C with the following antibodies: rabbit anti-collagen type I (dilution 1:1,000) and rabbit anti-fibronectin (1:1,000) from Chemicon International (Temecula, CA, United States); rabbit anti-ACVRL1 (ALK1) (1:1,000) from Abgent (Derio, Spain); mouse anti-*α*-SMA (1:5,000) from Sigma-Aldrich (Madrid, Spain); mouse anti-PCNA (1:1,000) from Transduction Laboratories (Madrid, Spain); and mouse anti-GAPDH (1:40000) from Ambion (Barcelona, Spain). After overnight incubation with the primary antibodies, membranes were incubated with the corresponding horseradish peroxidase-conjugated secondary antibodies during 60 min (1:10000) and were developed using ECL chemiluminescence reagent (Amersham Biosciences, Barcelona, Spain). Developed signals were recorded on X-ray films (Fujifilm Spain, Barcelona, Spain) for densitometric analysis (Scion Image software, Frederick, MD, United States). GAPDH (Ambion, Barcelona, Spain) was used as loading control. GAPDH was incubated in the same membrane that the protein of interest when it was possible.

### Histochemistry and Immunohistochemistry

3 µm sections were cut from paraffin-embedded samples and stained with haematoxylin-eosin, picrosirius, and Masson’s trichrome. Sirius red staining was evaluated by a quantitative scoring system, Fiji (https://imagej.net/software/fiji/), released as open source under the GNU General Public License in 12 randomly selected fields (200X) per experimental group.

Immunohistochemistry was performed on buffered formalin-fixed, paraffin-embedded tissues as previously described (Rodríguez-Peña et al., 2002; Grande et al., 2010) Briefly, 3 µm sections were deparaffinized in xylene and rehydrated in graded ethanols (100, 80, 70 and 50%) before antigen retrieval with sodium citrate buffer pH = 6.0, Then, primary antibodies were incubated overninght. Primary antibodies were: mouse anti-alpha smooth muscle actin (*α*-SMA, dilution 1:100, from Sigma-Aldrich), CD31 (Abcam) and rabbit anti-S100A4 (1:100 from Chemicon International, Temecula, CA, United States), mouse anti-VEGF (1:100 from Santa Cruz Biotechnology. After that staining continued with the peroxidase-antiperoxidase method. Tissue sections were incubated with the corresponding horseradish peroxidase-conjugated secondary antibodies during 60 min (1:250). After three washes in phosphate-buffered saline (PBS: 0.81% NaCl, 2.6 mM H_2_KPO_4_, 4.1 mM HNa_2_PO_4_), sections were sequentially incubated with the Novolink Polymer Detection System (Novocastra, Newcastle, United Kingdom) using 3,3′-diaminobenzidine (Biogenez, San Ramón CA, United States) as chromogen. Sections were counterstained with haematoxylin and were dehydrated and cover slipped. Endogenous peroxidase was blocked by incubation in 3% hydrogen peroxide. For an adequate optimization of the method, negative controls were prepared without primary antibodies. VEGF staining was evaluated using Fiji as mentioned above in 10–15 randomly selected fields (400X) per experimental group.

### Immunofluorescence Staining

Paraffin-embedded tissues were cut in 3 µm sections. Heat-induced antigen retrieval was performed in sodium citrate buffer pH 6.00 in a microwave owen, and washed with PBS. Sections were incubated with endomucin (Santa Cruz Biotechnology) in combination or not with anti-*α*-SMA (Sigma Aldrich) overnight at 4°. Following three washes in PBS, sections were incubated with anti-rat 488 Alexa and anti-mouse 546 (Molecular Probes, Barcelona, Spain), diluted 1:200 for 60 min at room temperature, washed in PBS, and stained with 2 µM Hoechst 33258 (Molecular Probes, Barcelona, Spain). Slides were rinsed in PBS and mounted in Prolong anti-fade (Invitrogen, Barcelona, Spain). Confocal images were photographed using a Zeiss Axiovert 200 M microscope and a Zeiss LSM 510 confocal module. All images were obtained with identical parameters for intensity, pinhole aperture, etc. Image manipulation of immunofluorescence analysis was performed using the same settings in all the samples and pictures shown in this manuscript, and following the image manipulation guidelines from the journal.

### Blood Vessel Density Analysis

Abundance of peritubular capillaries was assessed by counting the number of CD31 or endomucin-positive microvessels in peritubular areas in the kidney cortex across 5 different fields per kidney. Glomerular capillaries were not considered for the analysis.

### Vascular Rarefaction and Endothelial-Myofibroblast Transition Analysis

Apart from the blood vessel density analysis, vascular rarefaction was assessed by the individual quantitation of endomucin+ endothelial cells across 5 different fields per kidney (SO and O kidneys form *Alk1* ^*+/+*^ and *Alk1* ^*+/−*^ mice). Myofibroblast emergence was also assessed by the individual number of *α*-SMA+ cells excluding vascular smooth muscle cells. Hoechst counterstaining helped us to identify individual cells. Endothelial-myofibroblast transition was assessed by counting the double endomucin+ and α-smooth muscle actin *α*-SMA+ cells versus total endomucin + cells across 5 fields per kidney.

### Statistical Analysis

Data are expressed as mean ± standard error of the mean (SEM). The Kolmogorov-Smirnov test was used to assess the normality of the data distribution. Comparison of means was performed by two way analysis of variance (ANOVA) with Tukey’s HSC post hoc test. Data was analyzed using Graph Pad Prism software 9.0. A “p” value lower than 0.05 was considered statistically significant.

## Results

### Renal Injury and Kidney Fibrosis After 3 days UUO in *Alk1* ^*+/+*^ and *Alk1* ^*+/−*^ Mice

**FIGURE 1:**
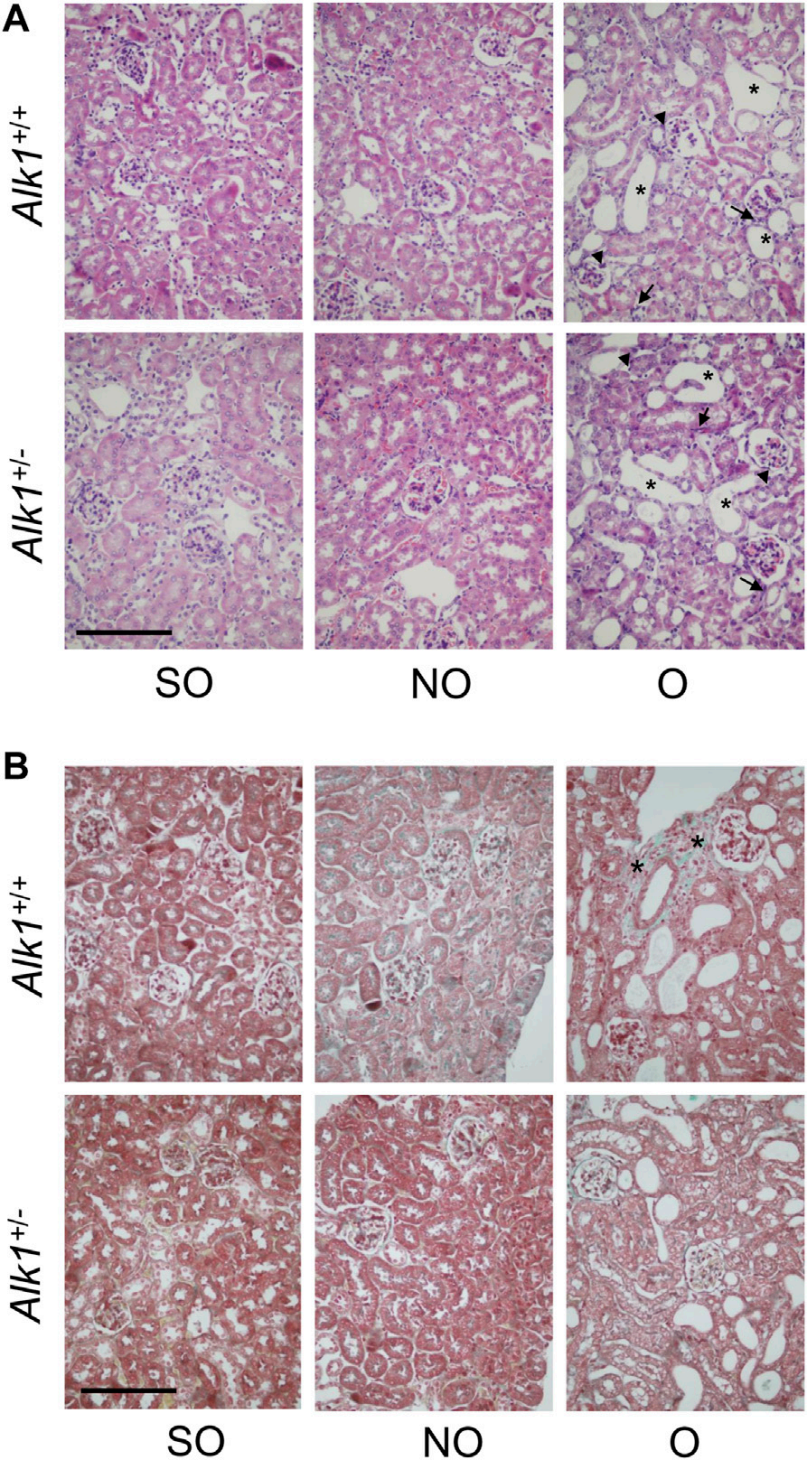
UUO modifies the renal ultrastructure after 3 days in *Alk1* ^*+/+*^ and *Alk1* ^*+/−*^ mice. **(A)** Haematoxylin-eosin staining in SO, NO and O kidneys from *Alk1* ^*+/+*^ and *Alk1* ^*+/−*^ mice show the typical features of the early stages of UUO such as tubular dilatation (asterisk), interstitial cell proliferation (arrow) or immune cell infiltration (arrowhead), being these features similar in O kidneys from *Alk1* ^*+/+*^ and *Alk1* ^*+/−*^ mice. **(B)** Masson’s trichrome staining in SO, NO and O kidneys from *Alk1* ^*+/+*^ and *Alk1* ^*+/−*^ mice showing lower ECM deposition (asterisk) in O kidneys from *Alk1* ^*+/−*^ *.* Scale bar = 200 microns in both panels.

O kidneys from both *Alk1* ^*+/+*^ and *Alk1* ^*+/−*^ mice show the histological events that take place after the UUO: Tubular cell injury, tubular dilation, inflammation; as shown with the Haematoxylin-eosin staining (Figure 1A) and tubule-interstitial fibrosis, as shown with the Masson’s trichrome staining (Figure 1B). While we observed no differences in kidney injury between both genotypes, we detected lower tubule-interstitial fibrosis in *Alk1* ^*+/−*^ mice.

### *Alk1* ^+/−^Mice Show Decreased Tubulo-Interstitial Fibrosis After 3 days of Unilateral Ureteral Obstruction (UUO)

**FIGURE 2:**
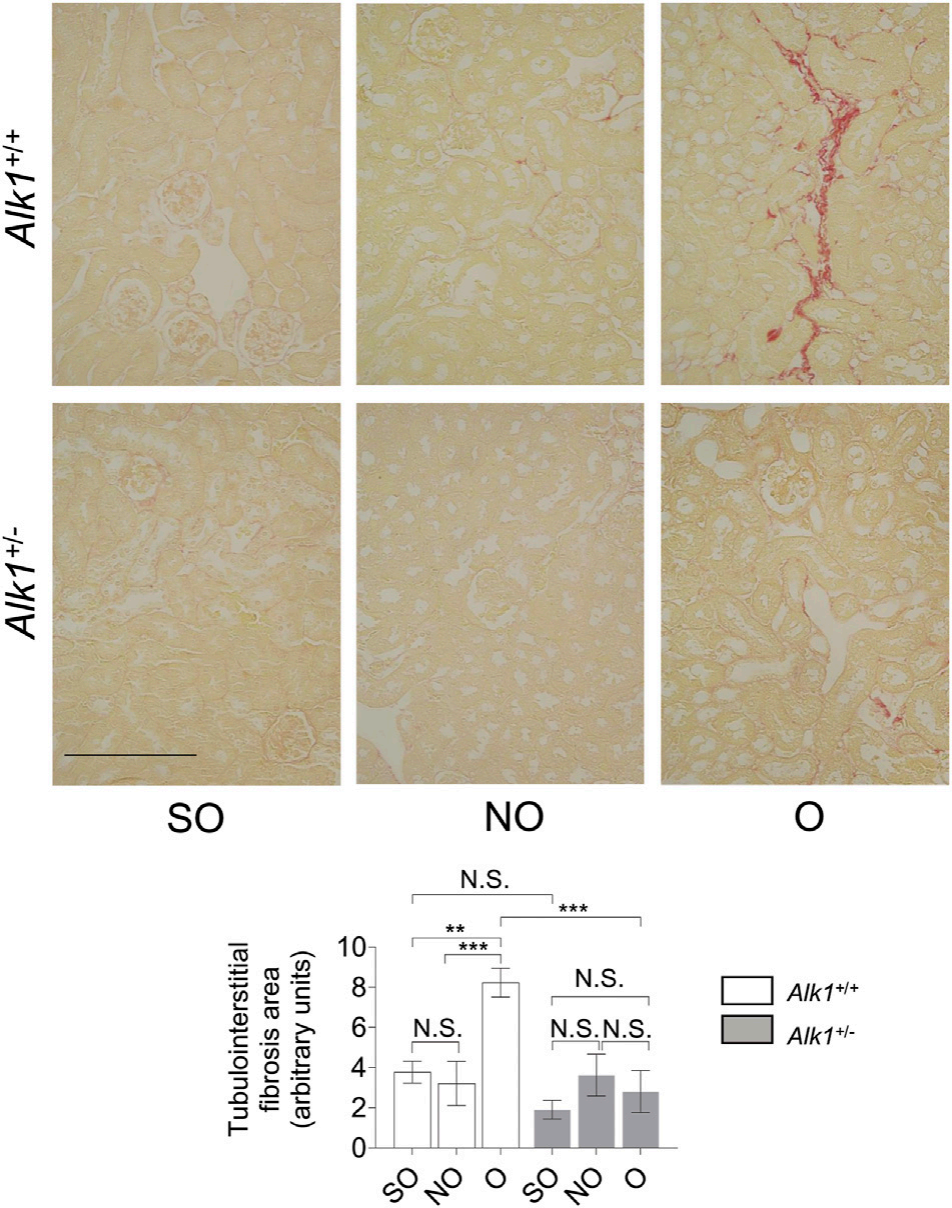
*Alk1* ^*+/−*^ mice show lower kidney fibrosis after 3 days UUO. Sirius red staining in SO, NO and O kidneys from *Alk1* ^*+/+*^ and *Alk1* ^*+/−*^ mice (*N* = 5) (upper panel). Analysis of tubule-interstitial fibrosis area in SO, NO and O kidneys from *Alk1* ^+/+^ and *Alk1* ^*+/−*^ mice, assessed by Fiji (ImageJ) software. **p < 0.01; ***p < 0.001 (Two-way ANOVA); N.S = Not statistically significant. Scale bar = 250 microns.

One of the most representative features of obstructive nephropathy is the accumulation of ECM proteins in the tubular interstitium, such as collagens (collagen I or collagen III), fibronectin or laminin. After our analysis of the picrosirius red staining, we observed increased levels of collagens in O kidneys form *Alk1* ^*+/+*^ mice but not in O kidneys from *Alk1* ^*+/−*^ mice (Figure 2).

**FIGURE 3:**
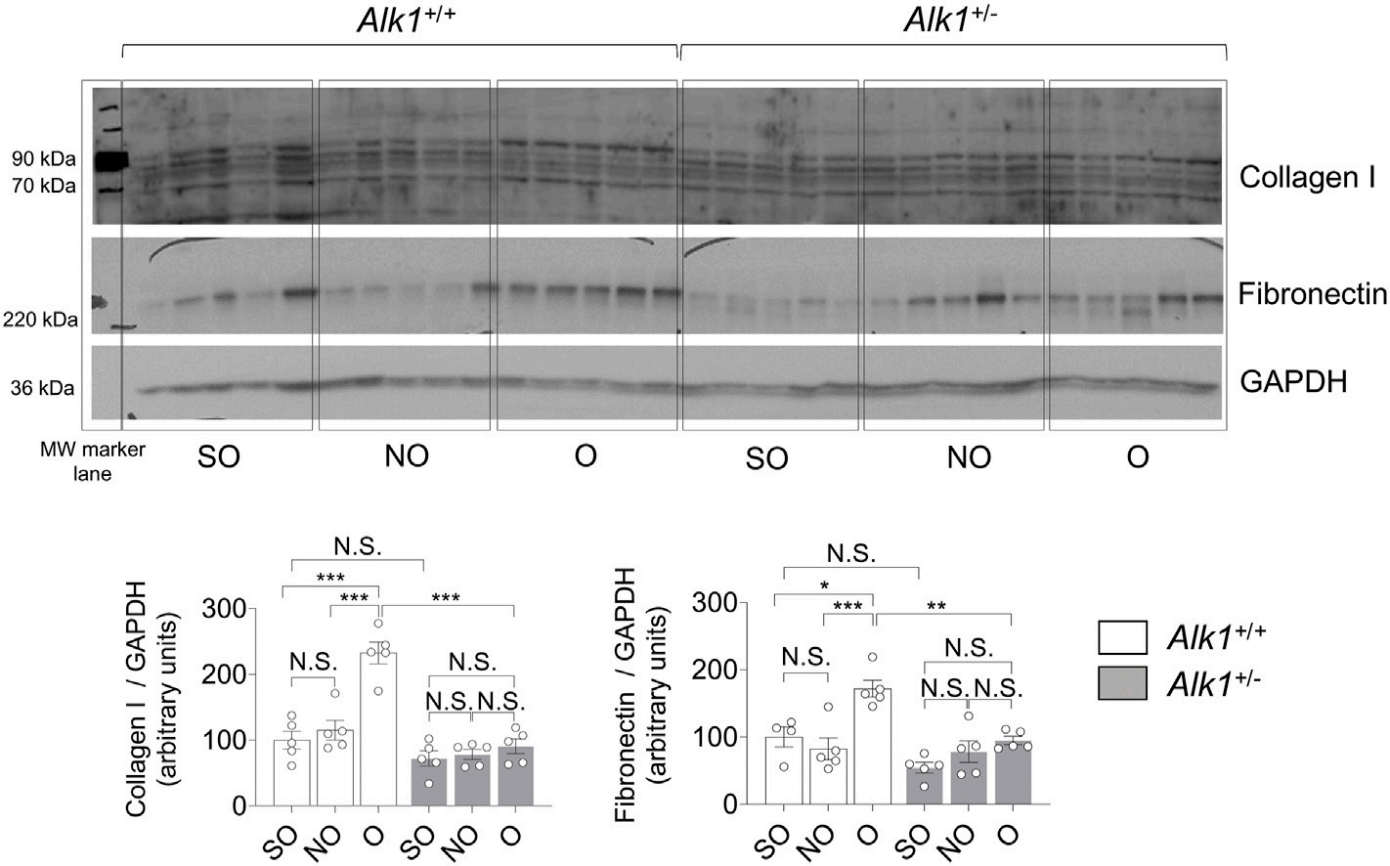
Obstructed kidneys from *Alk1* ^*+/−*^ mice show reduced ECM protein levels after 3 days UUO. Western blot analysis of collagen I and fibronectin protein expression in SO, NO and O kidneys from *Alk1* ^*+/+*^ and *Alk1* ^*+/−*^ mice, and quantification of the corresponding densitometry. Bars represent the ratio between the proteins and GAPDH, used as loading control. *p < 0.05; **p < 0.01; ***p < 0.001; N.S = Not statistically significant (Two-way ANOVA). One statistical outlier was removed from the analysis of fibronectin in a SO mice from *Alk1* ^*+/+*^ mice.

There is also an increase in the expression of ECM proteins (collagen I, fibronectin) in O kidneys from *Alk1* ^*+/+*^ but not in O kidneys from *Alk1* ^*+/−*^ mice evaluated by western blot (Figure 3).

### Reduced Renal Myofibroblast Emergence and Proliferation in *Alk1* ^*+/−*^ Mice

**FIGURE 4:**
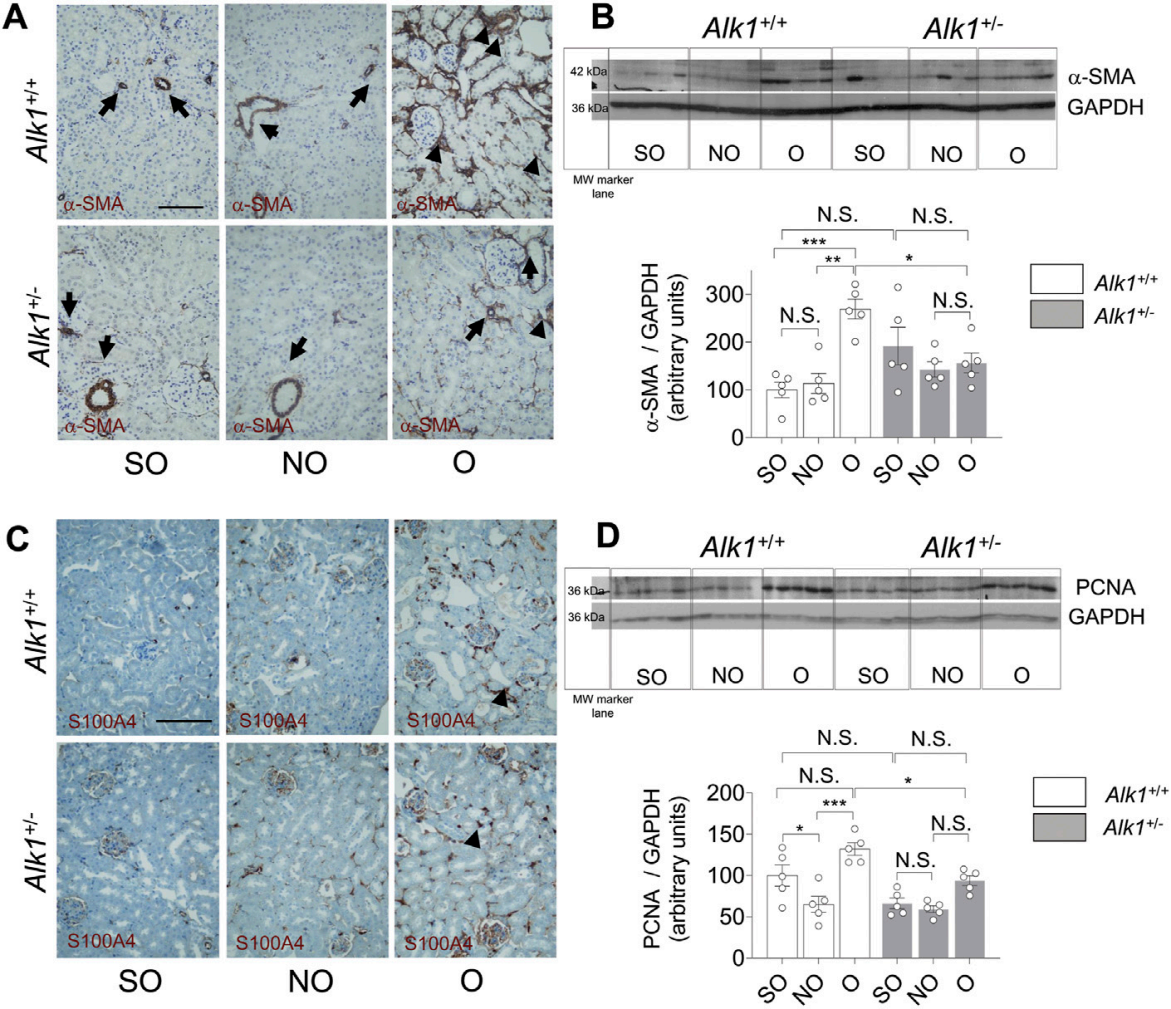
Reduced myofibroblast abundance and proliferation in obstructed kidneys from *Alk1* ^*+/−*^ mice after 3 days UUO. **(A)** *α*-SMA immunostaining in SO, NO and O kidneys from *Alk1* ^*+/+*^ and *Alk1* ^*+/−*^ mice. **(B)** Western blot analysis of *α*-SMA protein expression in SO, NO and O kidneys from *Alk1* ^*+/+*^ and *Alk1* ^*+/−*^ mice and quantification of the corresponding densitometry analysis (*N* = 5). Bars represent the ratio between *α*-SMA and GAPDH, used as loading control. **(C)** FSP1/S100A4 immunostaining in SO, NO and O kidneys from *Alk1* ^*+/+*^ and *Alk1* ^*+/−*^ mice. **(D)** Western blot analysis of PCNA protein expression in SO, NO and O kidneys from *Alk1* ^*+/+*^ and *Alk1* ^*+/−*^ mice and quantification of the corresponding densitometry analysis. Bars represent the ratio between PCNA and GAPDH, used as loading control. *p < 0.05; **p < 0.01; ***p < 0.001; N.S = Not statistically significant (Two-way ANOVA). The loading control for PCNA is the same as that used in Figure 3, as both Collagen I and PCNA were incubated in the same membrane. Arrows in **(A)** identify VSMCs. Arrowheads in **(A)** and **(B)** identify tubulo-interstitial myofibroblasts. Scale bar = 100 microns in A, 150 microns in **(C)**.

**FIGURE 5:**
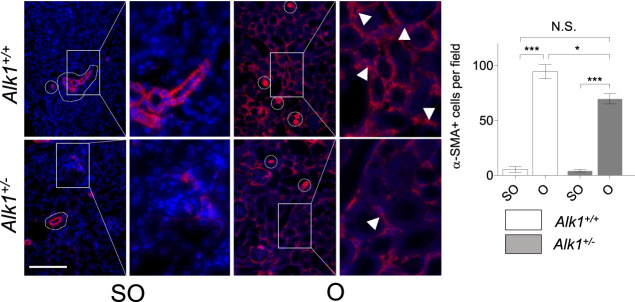
Analysis of *α*-SMA + myofibroblasts. Identification of *α*-SMA+ myofibroblasts by immunofluorescence of *α*-SMA with Hoechst counterstaining in SO and O kidneys from *Alk1* ^*+/+*^ and *Alk1* ^*+/−*^ mice. Squares identify zoomed areas *p < 0.05; ***p < 0.001; N.S = Not statistically significant. (Two-way ANOVA). Cropped areas identify *α*-SMA+ VSMCs from small vessels. Arrowheads identify *α*-SMA+ tubulo-interstitial myofibroblasts. Scale bar = 150 microns.

Renal myofibroblasts emerge and proliferate in the first steps of obstructive nephropathy. After 3 days of UUO we observe an increase in the presence of myofibroblasts in the tubular initerstitium of *Alk1* ^*+/+*^ mice, evaluated by immunostaining of the myofibroblast markers *α*-smooth muscle actin (*α*-SMA) (Figure 4A) and FSP1/S100A4 (Figure 4C) and by *α*-SMA western blot analysis (Figure 4B) in O kidneys from *Alk1* ^*+/+*^ mice. However, we barely observe those increases in O kidneys from *Alk1* ^*+/−*^ mice. (Figures 4A–C). As *α*-SMA is not only a specific maker for myofibroblasts, because it is highly expressed in vascular smooth muscle cells (VSMCs) we quantified the *α*-SMA+ individual cells by immunofluorescence with Hoechst counterstaining excluding VSMCs, and we show that O kidneys from *Alk1* ^*+/+*^ mice show a higher number of *α*-SMA+ myofibroblasts than O kidneys from *Alk1* ^*+/−*^ mice (Figure 5). In O kidneys from *Alk1* ^*+/−*^ mice the presence of *α*-SMA positive cells is reduced and correlates with the lower ECM deposition observed in these animals. Moreover, the increase in cell proliferation is lower in O kidneys from *Alk1* ^*+/−*^ mice, assessed by proliferating cell nuclear antigen (PCNA) expression (Figure 4D). Taken all these together, we can suggest that ALK1 heterozygosity leads to a lower kidney fibrosis due to a reduced abundance and proliferation of myofibroblasts.

### ALK1 Deficiency Ameliorates the Microvascular Damage Early Produced by UUO

As mentioned before, microvascular rarefaction is a feature of tubule-interstitial fibrosis and it contributes to the progression of hypoxia and tissue fibrosis. In the early stages of UUO, there is a vessel regression phase in which endothelial cells undergo apoptosis and pericyte adhesion is disrupted (Kida et al., 2014). Several studies show that ALK1 is involved in vessel maturation and quiescence (Akla et al., 2018; Viallard et al., 2020).

**FIGURE 6:**
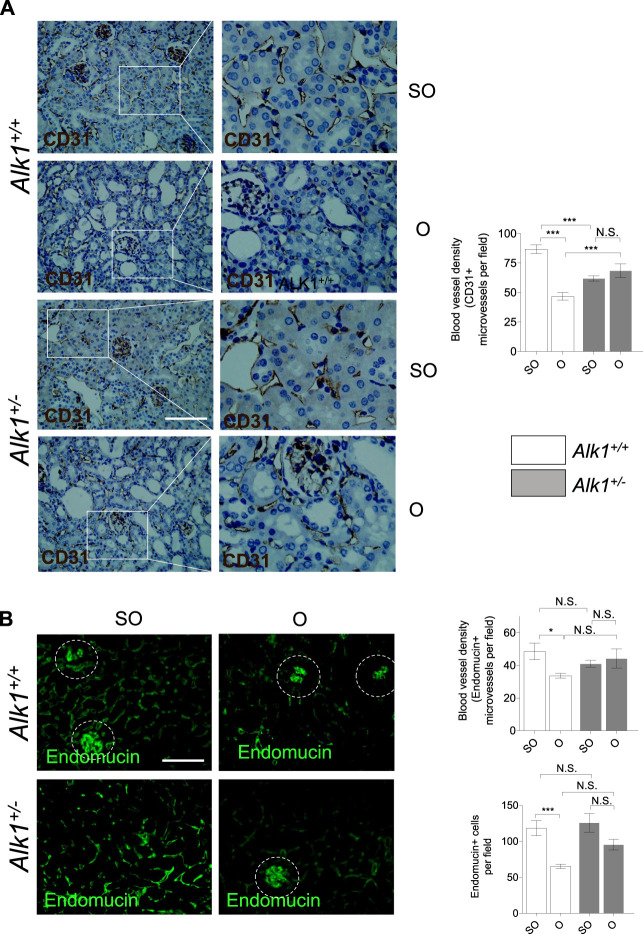
Impaired peritubular capillaries rarefaction in *Alk1* ^*+/−*^ mice. **(A)** CD31 immunostaining in SO and O kidneys from *Alk1* ^*+/+*^ and *Alk1* ^*+/−*^ mice and blood vessel density analysis, represented as CD31 ^+^ vessels per field. **(B)** Endomucin immunofluorescence staining in SO and O kidneys from *Alk1* ^*+/+*^ and *Alk1* ^*+/−*^ mice and blood vessel density quantification from endomucin staining, represented as microvessels per field (upper graph) and endomucin+ cells per field (lower graph) in SO and O kidneys from *Alk1* ^*+/+*^ and *Alk1* ^*+/−*^ mice. *p < 0.05; ***p < 0.001; N.S. Not statistically significant (Two-way ANOVA). Squares in **(A)** indicate the zoomed areas. Scale bar = 200 microns in both panels. Blood vessels from the glomeruli in **(B)** (highlighted as cropped áreas) were not counted.

We observe a decrease in blood vessel density in O kidneys from *Alk1* ^*+/+*^ mice, assessed by immunostaining of the endothelial markers CD31 (Figure 6A) and endomucin (Figure 6B), similar to that previously described in other studies performed in the UUO model (Kida et al., 2014). However, blood vessel density was maintained in *Alk1* ^*+/−*^ mice after UUO, suggesting that ALK1 heterozygosity protects from vascular rarefaction in the UUO early stages.

### Impaired Emergence of Myofibroblasts From Endothelial Cell Origin in *Alk1* ^*+/−*^ Mice

**FIGURE 7:**
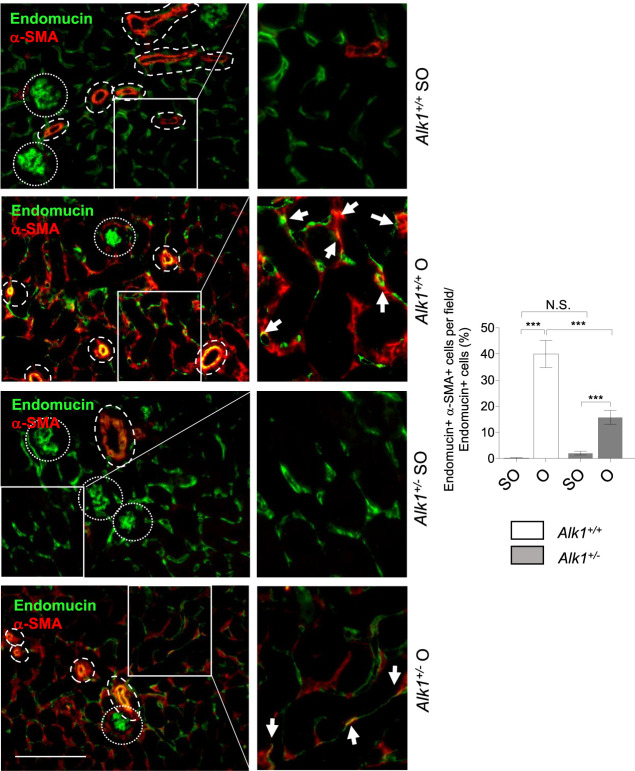
Endothelial-to-myofibroblast transdifferentiation after UUO in *Alk1* ^*+/+*^ and *Alk1* ^*+/−*^ mice. Double immunofluorescence of endomucin (endothelial marker) and *α*-SMA (myofibroblast marker) in SO and O kidneys from *Alk1* ^*+/+*^ and *Alk1* ^*+/−*^ mice and quantification of double endomucin and *α*-SMA positive cells. Cropped areas with small dashed line are glomeruli, not considered for the analysis. Cropped areas with large dashed lines are small vessels, also not considered for the analysis. ***p < 0.001; N.S. Not statistically significant (Two-way ANOVA). Arrows identify double endomucin+—*α*-SMA+ cells. Scale bar = 200 microns.

As stated before, myofibroblasts in the obstructed kidney emerge from different origins such as proliferating local resident fibroblasts, bone marrow derived cells or vascular cells. Vascular endothelial cells and pericytes can transdifferentiate into myofibroblasts. To dissect the myofibroblast cells that arise from endothelial cells we have double-immunostained kidney sections with an endothelial marker (endomucin) and a myofibroblast marker (*α*-SMA). Thus, cells with double positive staining for endomucin and *α*-SMA are endothelial cells being transdifferentiated into myofibroblasts. We observed that these double stained cells are more abundant in O kidneys from *Alk1* ^*+/+*^ mice than in O kidneys from *Alk1* ^*+/−*^ mice (Figure 7). This finding indicates that the lower abundance of myofibroblasts observed in ALK1 heterozygous mice is due to a lower transdifferentiation from endothelial cells and this correlates with the PTC stability after 3 days UUO in *Alk1* ^*+/−*^ mice.

### *Alk1* ^*+/−*^ Mice Are Protected From Vessel Regression After 3 days UUO

**FIGURE 8:**
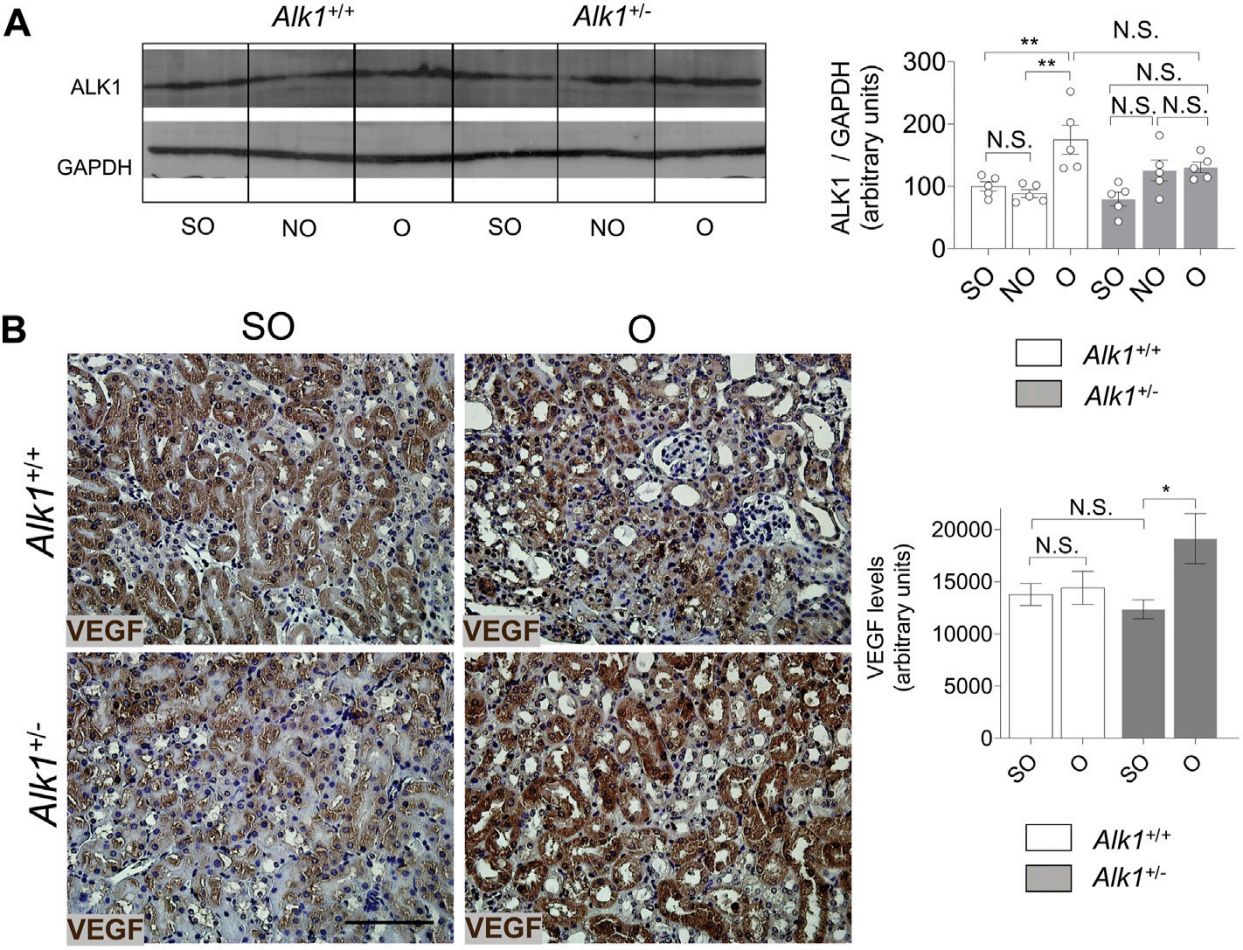
ALK1 and VEGF protein expression after UUO. **(A)** Western blot analysis of ALK1 protein in SO, NO and O kidneys from *Alk1* ^*+/+*^ and *Alk1* ^*+/−*^ mice (*N* = 5), and quantification of the corresponding densitometry analysis. Bars represent the ratio between the proteins and GAPDH, used as loading control. **(B)** VEGF immunohistochemistry representative pictures and quantification of VEGF levels (using Fiji software) in SO, NO and O kidneys from *Alk1* ^*+/+*^ and *Alk1* ^*+/−*^ mice. *p < 0.05; **p < 0.01 N.S. Non statistically significant (Two-way ANOVA). Scale bar = 150 microns.

ALK1 was described by David et al. (2008) as a molecule which induces quiescence and inhibits endothelial cell proliferation and migration (Lamouille et al., 2002; David et al., 2008). Angiogenesis is a process linked with the development of tubule-interstitial fibrosis after UUO. We found an increase of ALK1 expression in O kidneys from both *Alk1* ^*+/+*^ mice which may indicate the beginning of the regression phase of angiogenesis after obstruction. As expected, we do not observe increased levels of ALK1 receptor in *Alk1* ^*+/−*^ mice after UUO, suggesting a pro-angiogenic effect in these animals (Figure 8A). To elucidate the differences in the angiogenic process during vascular rarefaction after UUO, we have analyzed the levels of one of the most important angiogenic factors, vascular endothelial growth factor (VEGF). We detected no differences in VEGF expression after UUO in *Alk1* ^*+/+*^ mice but we observed a higher expression in O kidneys from *Alk1* ^*+/−*^ mice (Figure 8B). Taken all these together, we suggest that the lower ALK1 levels and increased levels of VEGF in O kidneys from *Alk1* ^*+/−*^ mice may correlate with a delay of the regression phase of angiogenesis that prevents endothelial-pericyte detachment, contributing to microvascular preservation and impaired endothelial to myofibroblast transdifferentiation.

## Discussion

Renal myofibroblasts are the main source of ECM proteins during tubule-interstitial fibrosis (LeBleu and Kalluri, 2011). Myofibroblasts are activated fibroblasts with high contractile capacity, and with a high capacity to synthesize ECM proteins such as collagens, fibronectin or laminin (Munoz-Felix and Martinez-Salgado, 2021). These cells emerge during the first steps of the fibrotic process from different origins. Numerous studies attributed their origin to the epithelial-to-mesenchymal transition (EMT) program (Grande and López-Novoa, 2009). However, although the EMT process has been validated in renal cells *in vitro* (Docherty et al., 2006a), the contribution of the epithelial components to myofibroblast abundance is very limited, as it has been demonstrated *in vivo* (Picard et al., 2008; Grande et al., 2015). The most important origins and mechanisms of myofibroblast emergence are proliferating local resident fibroblasts and the transdifferentiation from endothelial cells or and pericytes (LeBleu et al., 2013).

**FIGURE 9:**
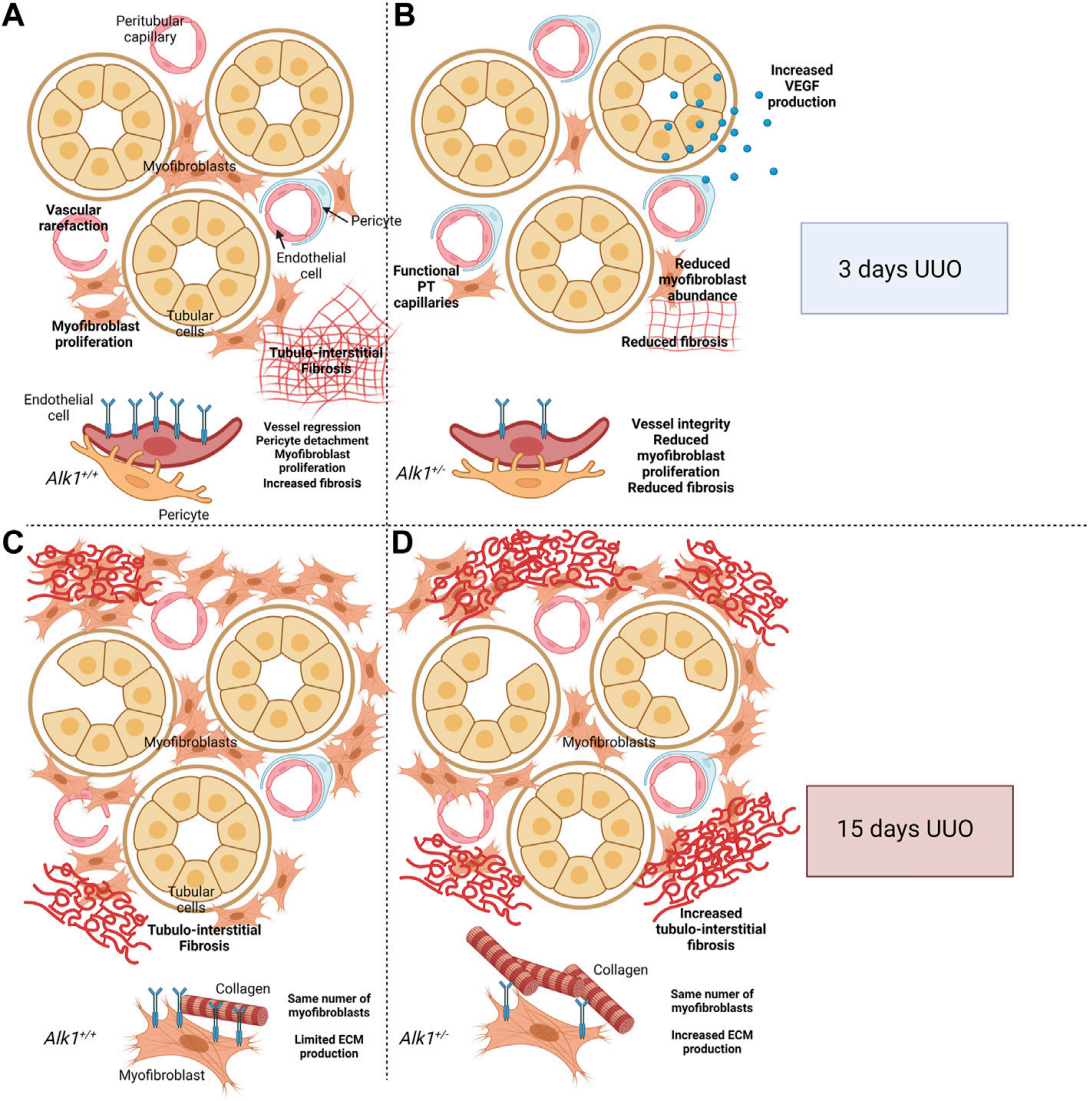
Proposed cellular mechanism. After 3 days of Unilateral Ureteral Obstruction (UUO), myofibroblasts emerge in the renal tubular interstitium and synthesize ECM proteins. At the same time, peritubular capillaries (PTC) undergo vascular rarefaction. This process starts with an angiogenic phase followed by a regression phase in which endothelial cells detach from pericytes and basement membrane, followed by apoptosis and leading to loss of functional capillaries. Both endothelial cells and pericytes can transdifferentiate into myofibroblasts and act as a source of extracellular matrix (ECM) components **(A,C)**. ALK1 heterozygosity is associated with PTC stability linked to an angiogenic process VEGF-dependent and the reduction of myofibroblast abundance, leading to reduced tubule-interstitial fibrosis **(A,B)** Previous results from our laboratory demonstrated that after 15 days of UUO, *Alk1* ^*+/+*^ and *Alk1* ^*+/−*^ mice show the same number of myofibroblasts but those from *Alk1* ^*+/−*^ mice produce higher amounts of ECM proteins leading to increased tubulointerstitial fibrosis **(C,D)**. Figure 9 was created using BioRender.com.

In this study we observe a lower kidney fibrosis after 3 days UUO which correlates with a lower myofibroblast abundance in *Alk1* ^*+/−*^ mice. Moreover, we show a lower microvascular rarefaction in these mice. Vascular rarefaction is involved in myofibroblast emergence by different mechanisms. In the early phases of UUO, endothelial cells undergo an apoptotic program which lead to the detachment of endothelial cells from pericytes (Kida et al., 2014). Endothelial cells can transdifferentiate into myofibroblasts *via* the endothelial to mesenchymal transition program (Zeisberg et al., 2008). On the other hand, pericytes can migrate from the basement membrane and transdifferentiate into myofibroblasts (Kida et al., 2014). Our data suggest that the maintenance of the microvascular architecture observed in *Alk1* ^*+/−*^ may be related with the lower emergence of myofibroblasts observed in these mice and the lower ECM deposition as a possible consequence of the reduced number of myofibroblasts (Figure 9).

The process by which PTC undergo rarefaction comprises two different stages: Initially, there is an angiogenic phase where angiogenic factors such as VEGF are upregulated and inflammatory cell infiltration happens. Later, the vascular regression phase occurs when a decrease of angiogenic factors and an increase of anti-angiogenic factors takes place in the obstructed kidney (Kida et al., 2014). Endothelial cells and pericytes are detached in the regression phase and can be transdifferentiated to myofibroblasts. Our data suggest that ALK1 is regulating this phenomenon. We observed lower vascular rarefaction in *Alk1* ^*+/−*^ mice after 3 days of UUO. ALK1 regulates negatively the activation phase of angiogenesis (Ayuso-Inigo et al., 2021) and it is expected that lower levels of ALK1 in *Alk1* ^*+/−*^ mice lead to a maintained angiogenic phase or an impaired vessel regression phase, which also correlates with the higher VEGF levels observed in O kidneys from *Alk1* ^*+/−*^ mice. Our observations are in concordance with those of Sharpfenecker et al. (2011), who demonstrated in a kidney fibrosis model after irradiation that *Alk1* ^*+/−*^ mice show lower vascular injury after 20 weeks of irradiation, and this correlated with higher levels of VEGF and VEGFR2 at that time point (Scharpfenecker et al., 2011).

Years ago, we demonstrated a role of ALK1 in counteracting TGF-*β*1-induced kidney fibrosis at 15 days UUO (Muñoz-Félix et al., 2014a). In that context, both *Alk1* ^*+/−*^ and *Alk1* ^*+/+*^ mice showed same myofibroblast abundance but *Alk1* ^*+/−*^ myofibroblasts produced higher amounts of ECM proteins (Muñoz-Félix et al., 2014b; Oujo et al., 2014). In this manuscript we demonstrate that after 3 days UUO ALK1 heterozygosity is associated with a lower myofibroblast emergence, due to a higher microvessel stability, and this lower number of myofibroblasts results in a decrease in tubulo-interstitial fibrosis. We suggest that in the early stages of UUO, ALK1 function is mainly related with its effect on endothelial cells. The different effects of ALK1 receptor in kidney fibrosis at different time points following UUO can be explained by the different cellular players in these different stages of fibrosis progression. We suggest that in the early stages of the ureteral obstruction ALK1 is regulating the myofibroblast emergence from endothelial cells while after 15 days UUO the fibrotic program is completely established and myofibroblast number is elevated and ALK1 regulates negatively ECM protein synthesis by myofibroblasts through an inhibition of TGF-*β*1/Smad2/3 pathway (Muñoz-Félix et al., 2014b).

Although our previous studies demonstrate a role of ALK1 in regulating ECM production by ECM producing cells like fibroblasts, the biological role of ALK1 has been traditionally considered more relevant in the regulation of endothelial cell balance during development, cardiovascular diseases and tumor angiogenesis (Ayuso-Inigo et al., 2021). To understand ALK1 function in tissue fibrosis is very important to consider two molecular players that regulate ALK1 activity: Its high affinity ligand BMP9 (David et al., 2007b) and the coreceptor endoglin (Lebrin et al., 2004; López-Novoa and Bernabeu, 2010). Both molecules have been studied in depth as regulators of vascular homeostasis and tissue fibrosis (Table 1).

**TABLE 1:** ALK1, BMP9 and Endoglin effects in tissue fibrosis.

ALK1				BMP9				Endoglin			
Profibrotic effect		Antifibrotic effect		Profibrotic effect		Antifibrotic effect		Profibrotic effect		Antifibrotic effect	
Ref.	Experimental model	Ref.	Experimental model	Ref.	Experimental model	Ref.	Experimental model	Ref.	Experimental model	Ref.	Experimental model
Scharpfenecker et al. (2011)	Kidney fibrosis by irradiation in *Alk1* ^*+/−*^ mice	Muñoz-Félix et al. (2014a)	Unilateral Ureteral Obstruction (UUO) during 15 days in *Alk1* ^*+/−*^ mice	Muñoz-Félix et al. (2016a)	Cultured mouse embryo fibroblasts	Morine et al. (2018)	Transverse aortic constriction (TAC) in Bmp9-KO mice.	Scharpfenecker et al. (2012)	Kidney fibrosis by irradiation in *Eng* ^*+/−*^ mice	Muñoz-Félix et al. (2016b)	UUO in *S-Eng* ^*+*^ mice (mice overexpressing human Short endoglin).
Morine et al. (2017b)	Deletion of ALK1 with conditional knockout mice	Morine et al. (2017a)	Transverse aortic constriction (TAC) in *Alk1* ^*+/−*^ mice	Li et al. (2018)	CCl_4_ induced liver fibrosis Bile duct ligation (BDL) induced liver fibrosis	Desroches-Castan et al. (2019a)	*Bmp9*-KO mice	Scharpfenecker et al. (2009)	Kidney fibrosis by irradiation in *Eng* ^*+/−*^ mice	Pericacho et al. (2013)	Cutured dermal fibroblasts from *Eng* ^*+/−*^ mice
Wiercinska et al. (2006)	Cultured hepatic stellate cells	Muñoz-Félix et al. (2014b)	Cultured mouse embryo fibroblasts form *Alk1* ^*+/−*^ mice	Breitkopf-Heinlein et al. (2017)	CCL_4_ and LPS induced liver fibrosis. BMP9 inactivated with adenoviruses	Desroches-Castan et al. (2019b)	*Bmp9*-KO mice	Scharpfenecker et al. (2013)	Kidney fibrosis by irradiation in *Eng* ^*+/−*^ mice	Velasco et al. (2008)	Cultured L6E9 rat myoblasts overexpressing L-Endoglin
Breitkopf-Heinlein et al. (2017)	CCL_4_ and LPS induced liver fibrosis. BMP9 inactivated with adenoviruses	Finnson et al. (2008)	Cultured human chondrocytes			Jiang et al. (2021)	Bleomycin-induced pulmonary fibrosis	Docherty et al. (2006b)	Kidney fibrosis induced by Ischaemia-reperfusion injury in *Eng* ^*+/−*^ mice	Finnson et al. (2010)	Cultured human chondrocytes
This current manuscript	UUO during 3 days in *Alk1* ^*+/−*^ mice					Chen et al. (2017)	BMP9 treatment in neonatal rats	Kapur et al. (2012)	TAC in *Eng* ^*+/−*^ mice	Alzahrani et al. (2018)	Skin fibrosis induced by bleomycin
								Oujo et al. (2014)	UUO in *L-Eng* ^*+*^ mice (mice overexpressing human Large endoglin).	Obreo et al. (2004)	Cultured L6E9 myoblasts
								Gerrits et al. (2020)	cultured human renal myofibroblasts	Diez-Marques et al. (2002)	Cultured human mesangial cells
								Owen et al. (2020)	Patients with cirrhosis	Rodríguez-Barbero et al. (2006)	Cultured L6E9 myoblasts
								Morris et al. (2011)	Cultured scleroderma (SSc) fibroblasts		
								Meurer et al. (2011)	Cultured Hepatic stellate cells		

However, the link of the ALK1-mediated endothelial effects and tissue fibrosis has not been studied in depth so far. Nevertheless, new functions have been recently described in different tissues such as liver and kidney. In the liver, ALK1 is involved in capillary fenestration and prevents the development of liver fibrosis (Desroches-Castan et al., 2019a). In this study, the authors show that mice lacking BMP9, a high affinity receptor for ALK1, show enlarged sinusoidal vessels and a reduced number of fenestrae. This suggests an interesting role of the BMP9-ALK1 axis in liver fibrosis protection. In renal tissue, a role for ALK1 in vascular cells has been described in diabetic nephropathy. ALK1 levels decrease in diabetic mice, being ALK1 expression circumscribed to glomerular capillaries. ALK1 heterozygous mice display albuminuria, as a result of changes in endothelial cells and podocytes, leading to exacerbated levels of collagen IV and thickening of the glomerular basement membrane (Lora Gil et al., 2020).

Our study shows that ALK1 is involved in the regulation of the stability of renal peritubular capillaries. In the same circumstances we observe a lower number of myofibroblasts in mice with lower expression of ALK1, which also show lower tubule-interstitial fibrosis. With these results, we suggest that both processes may be linked. Reduced levels of ALK1 together with an increase in VEGF levels maintains the stability of peritubular capillaries protecting kidney from myofibroblast emergence and ECM deposition. Considering all these facts, ALK1 seems to regulate the endothelial activation and quiescence in the context of UUO. Endothelial activation in kidney fibrosis occurs in the early steps of vascular rarefaction, and it is accompanied of endothelial and pericyte detachments. Both cell types might be the source of the increased number of myofibroblasts, as it has been demonstrated during the last years (Zeisberg and Kalluri, 2013).

## Conclusion

ALK1 is involved in the early changes of UUO, promoting the development of vascular rarefaction. *Alk1* ^*+/−*^ mice maintain the stability of the peritubular capillaries network after UUO, leading to a decrease of myofibroblasts emergence and ECM deposition.

## Data Availability

The raw data supporting the conclusion of this article will be made available by the authors, without undue reservation.
